# Di-μ-oxido-bis­({(*R*)-(–)-2-[1-(2-amino­propyl­imino)eth­yl]-1-naphtholato-κ^3^ *N*,*N*′,*O*}oxidovanadium(V))

**DOI:** 10.1107/S160053680801787X

**Published:** 2008-06-21

**Authors:** Grzegorz Romanowski, Artur Sikorski, Andrzej Wojtczak

**Affiliations:** a University of Gdańsk, Faculty of Chemistry, Sobieskiego 18/19, 80-952 Gdańsk, Poland; b Nicholas Copernicus University, Faculty of Chemistry, Gagarina 7, 87-100 Toruń, Poland

## Abstract

In the title dinuclear compound, [V_2_(C_15_H_17_N_2_O)_2_O_4_], each V^V^ atom is six-coordinated by one oxide group, and by two N and one O atom of the tridentate Schiff base ligand, and bridged by two additional oxide O atoms, resulting in a centrosymmetric dimer. The metal centre has a distorted octa­hedral coordination with the monoanionic Schiff base ligand occupying one equatorial and two axial coordination positions. The separation between V atoms is 3.214 (3) Å. In the crystal structure, there are N—H⋯O, C—H⋯O and C—H⋯π hydrogen bonds, and π–π inter­actions.

## Related literature

For general background, see: Sigel & Sigel (1995 ▶); Butler & Walker (1993 ▶); Martinez *et al.* (2001 ▶); Rehder (1991 ▶); Thompson & Orvig (2000 ▶); Evangelou (2002 ▶); Kwiatkowski *et al.* (2003 ▶, 2006 ▶, 2007 ▶); Romanowski *et al.* (2008 ▶); Rehder (1999 ▶); Colpas *et al.* (1994 ▶); Li *et al.* (1988 ▶); Fulwood *et al.* (1995 ▶). For related structures, see: Root *et al.* (1993 ▶); Romanowski *et al.* (2008 ▶); Rayati *et al.* (2007 ▶, 2008 ▶); Kwiatkowski *et al.* (2007 ▶). For the synthesis, see: Kwiatkowski *et al.* (2003 ▶). 
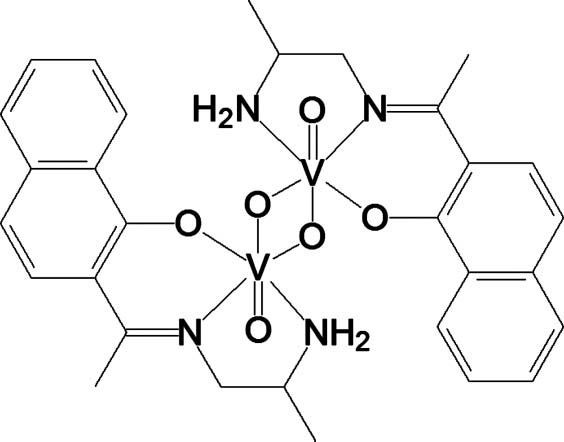


## Experimental

### 

#### Crystal data

[V_2_(C_15_H_17_N_2_O)_2_O_4_]
*M* *_r_* = 648.49
Monoclinic, 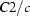

*a* = 25.187 (5) Å
*b* = 7.663 (2) Å
*c* = 16.898 (3) Å
β = 118.09 (3)°
*V* = 2877.3 (13) Å^3^
*Z* = 4
Mo *K*α radiation
μ = 0.70 mm^−1^
*T* = 298 (2) K
0.28 × 0.13 × 0.12 mm

#### Data collection

Oxford Diffraction Sapphire CCD diffractometer
Absorption correction: numerical (*CrysAlis RED*; Oxford Diffraction, 2006 ▶) *T*_min_ = 0.828, *T*_max_ = 0.918
9202 measured reflections
2474 independent reflections
2086 reflections with *I* > 2σ(*I*)
*R*_int_ = 0.078

#### Refinement

*R*[*F* ^2^ > 2σ(*F* ^2^)] = 0.084
*wR*(*F* ^2^) = 0.153
*S* = 1.23
2474 reflections
202 parameters
H-atom parameters constrained
Δρ_max_ = 0.66 e Å^−3^
Δρ_min_ = −0.41 e Å^−3^

### 

Data collection: *CrysAlis CCD* (Oxford Diffraction, 2006 ▶); cell refinement: *CrysAlis RED* (Oxford Diffraction, 2006 ▶); data reduction: *CrysAlis RED*; program(s) used to solve structure: *SHELXS97* (Sheldrick, 2008 ▶); program(s) used to refine structure: *SHELXL97* (Sheldrick, 2008 ▶); molecular graphics: *ORTEPII* (Johnson, 1976 ▶); software used to prepare material for publication: *SHELXL97* and *PLATON* (Spek, 2003 ▶).

## Supplementary Material

Crystal structure: contains datablocks global, I. DOI: 10.1107/S160053680801787X/xu2429sup1.cif

Structure factors: contains datablocks I. DOI: 10.1107/S160053680801787X/xu2429Isup2.hkl

Additional supplementary materials:  crystallographic information; 3D view; checkCIF report

## Figures and Tables

**Table 1:** Hydrogen-bond geometry (Å, °) *Cg*1 is the centroid of the C6-C9/C14/C15 ring.

*D*—H⋯*A*	*D*—H	H⋯*A*	*D*⋯*A*	*D*—H⋯*A*
N1—H1*A*⋯O1^i^	0.90	2.19	3.011 (6)	151
C7—H7*A*⋯O2^ii^	0.93	2.41	3.335 (7)	173
C18—H18*B*⋯O16^ii^	0.96	2.56	3.482 (8)	161
C3—H3*B*⋯*Cg*1^ii^	0.97	2.95	3.874 (7)	159

Symmetry codes: (i) 
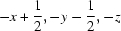
; (ii) 
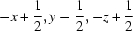
.

**Table 2:** π–π interactions (Å,°)

*CgI*	*CgJ*	*Cg*⋯*Cg*	Dihedral angle	Interplanar distance	Offset
*Cg*2	*Cg*2^iii^	3.518 (4)	0.0	3.365 (4)	1.025 (4)

Symmetry code: (iii) 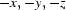. Notes: *Cg*2 represents the centroid of the C14–C19 ring. *Cg*⋯*Cg* is the distance between ring centroids. The dihedral angle is that between the planes of the rings *CgI* and *CgJ*. The interplanar distance is the perpendicular distance of *CgI* from ring *J*. Offset is the lateral offset distance of ring *I* from ring *J*.

**Fig. 1.**
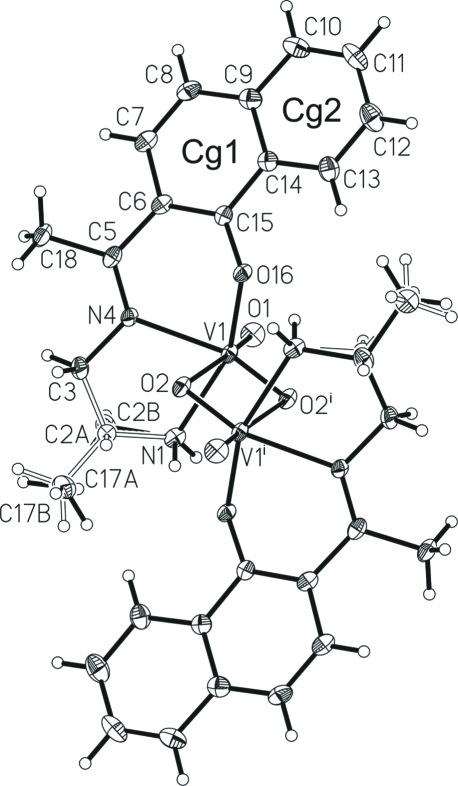
The molecular structure of the title compound, showing the atom-labelling scheme. Displacement ellipsoids are drawn at the 25% probability level and H atoms are shown as small spheres of arbitrary radius.

**Fig. 2.**
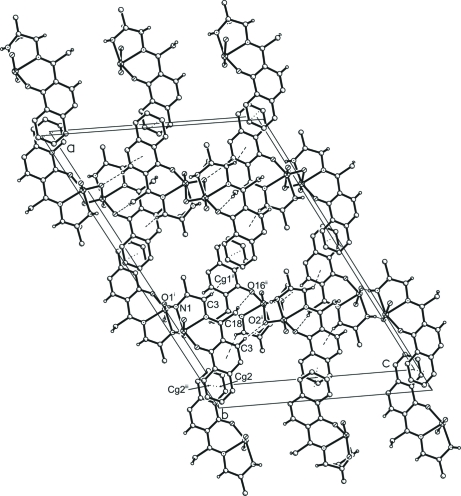
The arrangement of the molecules in the crystal structure viewed approximately along the c axis. The N—H···O, C—H···O and C—H···π interactions are represented by dashed lines and π-π interactions by dotted lines. H atoms not involved in interactions have been omitted. [Symmetry codes: (i) 1/2 - x, -1/2 - y, -z, 1/2 - z; (ii) 1/2 - x, -1/2 + y, 1/2 - z; (iii) -x, -y, -z].

## supplementary crystallographic information

### Comment

Vanadium is a trace element in diverse living forms (Sigel & Sigel, 1995). It plays active roles in many biologically important reactions such as halogenation of organic substrates, activation or fixation of nitrogen through an alternative pathway (Butler & Walker, 1993; Martinez *et al.*, 2001) and potent inhibitor of phosphate-metabolizing enzymes (Rehder, 1991). Some of the vanadium compounds stimulate glucose uptake and inhibit lipid breakdown in a manner remarkably reminiscent of insulin effects (Thompson & Orvig, 2000) or exert preventive effects against chemical carcinogenesis on animals (Evangelou, 2002). Recently, it has been established that vanadium(V) complexes with Schiff bases, which are excellent models for active sites of vanadium containing haloperoxidases, are able to catalyze the oxidation of organic sulfides to the corresponding sulfoxides (Kwiatkowski *et al.*, 2003, 2007; Romanowski *et al.*, 2008). A collection of such models discussed in some detail in a review (Rehder, 1999) show that they contained either N_2_O_2_ or NO_4–5_ set of donor atoms in the coordination sphere.

The half of the molecule, constituting the asymmetric part of the structure, is related to the other half by the center of symmetry (Fig. 1). The geometry of the coordination environment resembles two edge shared octahedrons that are significantly distorted. The V1=O1 bond length of 1.612 (4) Å is typically for the distances between vanadium and the doubly bonded oxygen atoms which are not involved in donor-acceptor interactions (Kwiatkowski *et al.*, 2003, 2006, 2007; Romanowski *et al.*, 2008). The O2, V1, O2^i^, V1^i^ atoms are situated in edges of a parallelogram with the acute O2-V1-O2^i^ angle of 77.09 (18)° [symmetry code: (i) -x+1/2,-y+1/2,-z]. The tridentate ligand is coordinated meridionally, its oxygen (O16) and primary amine nitrogen (N1) occupy axial positions. The V1—O1 bond is shorter than V1—O2^i^ bond (1.658 Å) due to involvement of O2^i^ atom in V1···V1^i^ bridging. The O1-V1-O2^i^ angle of 107.6 (2)° indicate significant double bond character of this bond (Colpas *et al.*, 1994) and is close to other *cis*-VO_2_ units (Li *et al.*, 1988). The five-membered ring comprising the propylenediamine moiety exhibits twofold disorder. A disorder of two carbon atoms in the aliphatic five-membered ring is interpreted assuming the presence of two conformations of the CH_2_—CH(CH_3_) fragment. The C2 and C17 atoms are disordered over two sites, with occupancy factors of 0.54 (2) and 0.46 (2) for C2A/C17A and C2B/C17B, respectively. The methyl group of the aliphatic five-membered ring assumes a pseudoequatorial position for both conformers. The ligand sites are diastereotopic and therefore the crystal of the complex may be considered as a solid solution of two covalent diastereomers (Kwiatkowski *et al.*, 2006). A rare case of two diastereomers in one crystal was demonstrated earlier (Fulwood *et al.*, 1995), in which is resolved the crystal structure of the monooxovanadium(V) Schiff base complex [VO(sal-*L*-ala)Bu*^s^*]Bu*^s^*OH. Structures of dimeric vanadium(V) Schiff base complexes, but derived from racemic 1,2-diaminopropane, have already been reported (Root *et al.*, 1993; Rayati *et al.*, 2007, 2008).

Hydrogen bonds, C—H···π and π-π interactions stabilize a network formed with the dimeric molecules (Fig. 2, Table 1, 2 and 3).

### Experimental

The title complex were obtained in a template/complexation reactions analogous to those described for preparation of dioxovanadium(V) complexes with Schiff base ligands (Kwiatkowski *et al.*, 2003). A sample of 10 mmol of *R*(-)-1,2-diaminopropane in 10 ml of absolute ethanol was added with stirring to a freshly filtered solution of vanadium(V) oxytriethoxide (10 mmol) in 50 ml of absolute ethanol producing a yellow suspension. 1-Hydroxy-2-acetonaphthone (10 mmol) dissolved in 10 ml of absolute ethanol was slowly added. After refluxing of the resulting mixture for 10 h and its cooling to room temperature the separated solid was filtered off and washed. Crystals suitable for X-ray analysis were obtained by slow recrystallization from ethanol/DMSO solution.

### Refinement

All H atoms were positioned geometrically and refined using a riding model, with C–H distances of 0.93–0.97 Å and with *U*_iso_(H) = 1.2*U*_eq_(C) (C–H = 0.96 Å and *U*_iso_(H) = 1.5*U*_eq_(C) for the methyl group) and with N–H distances of 0.90 Å and with *U*_iso_(H) = 1.2*U*_eq_(C). The C2 and C17 atoms are disordered over two sites, the occupancy ratio was refined and converged to 0.54 (2):0.46 (2).

### Crystal data

**Table:**

[V_2_(C_15_H_17_N_2_O)_2_O_4_]	*F*_000_ = 1344
*M**_r_* = 648.49	*D*_x_ = 1.497 Mg m^−^^3^
Monoclinic, *C*2/*c*	Mo *K*α radiation λ = 0.71073 Å
Hall symbol: -C 2yc	Cell parameters from 2875 reflections
*a* = 25.187 (5) Å	θ = 2.8–25.0º
*b* = 7.663 (2) Å	µ = 0.70 mm^−^^1^
*c* = 16.898 (3) Å	*T* = 298 (2) K
β = 118.09 (3)º	Needle, yellow
*V* = 2877.3 (13) Å^3^	0.28 × 0.13 × 0.12 mm
*Z* = 4	

### Data collection

**Table:**

Oxford Diffraction Sapphire CCD diffractometer	2474 independent reflections
Radiation source: fine-focus sealed tube	2086 reflections with *I* > 2σ(*I*)
Monochromator: graphite	*R*_int_ = 0.078
*T* = 298(2) K	θ_max_ = 25.0º
θ/2θ scans	θ_min_ = 2.8º
Absorption correction: numerical(CrysAlis RED; Oxford Diffraction, 2006)	*h* = −25→29
*T*_min_ = 0.828, *T*_max_ = 0.918	*k* = −9→8
9202 measured reflections	*l* = −20→20

### Refinement

**Table:**

Refinement on *F*^2^	Secondary atom site location: difference Fourier map
Least-squares matrix: full	Hydrogen site location: inferred from neighbouring sites
*R*[*F*^2^ > 2σ(*F*^2^)] = 0.084	H-atom parameters constrained
*wR*(*F*^2^) = 0.153	*w* = 1/[σ^2^(*F*_o_^2^) + (0.0144*P*)^2^ + 24.507*P*] where *P* = (*F*_o_^2^ + 2*F*_c_^2^)/3
*S* = 1.23	(Δ/σ)_max_ = 0.001
2474 reflections	Δρ_max_ = 0.66 e Å^−^^3^
202 parameters	Δρ_min_ = −0.41 e Å^−^^3^
Primary atom site location: structure-invariant direct methods	Extinction correction: none

### Special details

**Table:**

Geometry. All e.s.d.'s (except the e.s.d. in the dihedral angle between two l.s. planes) are estimated using the full covariance matrix. The cell e.s.d.'s are taken into account individually in the estimation of e.s.d.'s in distances, angles and torsion angles; correlations between e.s.d.'s in cell parameters are only used when they are defined by crystal symmetry. An approximate (isotropic) treatment of cell e.s.d.'s is used for estimating e.s.d.'s involving l.s. planes.

### Fractional atomic coordinates and isotropic or equivalent isotropic displacement parameters (Å^2^)

**Table:**

	*x*	*y*	*z*	*U*_iso_*/*U*_eq_	Occ. (<1)
V1	0.22582 (5)	0.07588 (12)	0.02520 (6)	0.0261 (3)	
O1	0.1947 (2)	−0.1142 (5)	0.0042 (3)	0.0419 (11)	
N1	0.3137 (2)	−0.0142 (6)	0.0585 (3)	0.0323 (12)	
H1A	0.3102	−0.1053	0.0226	0.039*	
H1B	0.3330	0.0713	0.0457	0.039*	
O2	0.28370 (18)	0.3407 (5)	0.0711 (2)	0.0308 (9)	
C3	0.3353 (3)	0.0368 (9)	0.2130 (4)	0.0413 (16)	
H3A	0.3546	0.1468	0.2393	0.050*	
H3B	0.3476	−0.0479	0.2612	0.050*	
N4	0.2701 (2)	0.0595 (6)	0.1714 (3)	0.0266 (10)	
C5	0.2442 (3)	0.0737 (7)	0.2207 (4)	0.0307 (13)	
C6	0.1780 (3)	0.0906 (7)	0.1799 (4)	0.0301 (13)	
C7	0.1481 (3)	0.0356 (7)	0.2291 (4)	0.0361 (15)	
H7A	0.1702	−0.0160	0.2850	0.043*	
C8	0.0877 (3)	0.0571 (9)	0.1958 (4)	0.0413 (16)	
H8A	0.0691	0.0174	0.2287	0.050*	
C9	0.0529 (3)	0.1389 (8)	0.1121 (4)	0.0384 (15)	
C10	−0.0103 (3)	0.1660 (9)	0.0753 (5)	0.0475 (18)	
H10A	−0.0300	0.1282	0.1069	0.057*	
C11	−0.0422 (3)	0.2461 (10)	−0.0052 (6)	0.056 (2)	
H11A	−0.0834	0.2628	−0.0275	0.068*	
C12	−0.0147 (3)	0.3033 (10)	−0.0545 (5)	0.056 (2)	
H12A	−0.0370	0.3601	−0.1089	0.067*	
C13	0.0463 (3)	0.2751 (8)	−0.0219 (4)	0.0415 (16)	
H13A	0.0646	0.3107	−0.0558	0.050*	
C14	0.0811 (3)	0.1941 (7)	0.0611 (4)	0.0315 (13)	
C15	0.1446 (2)	0.1620 (7)	0.0945 (4)	0.0264 (12)	
O16	0.16869 (17)	0.2114 (5)	0.0436 (2)	0.0288 (9)	
C18	0.2780 (3)	0.0802 (9)	0.3211 (4)	0.0414 (15)	
H18A	0.3139	0.1484	0.3397	0.062*	
H18B	0.2887	−0.0361	0.3444	0.062*	
H18C	0.2531	0.1324	0.3436	0.062*	
C2A	0.3524 (5)	−0.070 (2)	0.1537 (8)	0.042 (4)	0.54 (2)
H2A	0.3435	−0.1922	0.1590	0.051*	0.54 (2)
C17A	0.419 (2)	−0.055 (6)	0.180 (3)	0.067 (15)	0.54 (2)
H17A	0.4422	−0.1162	0.2359	0.101*	0.54 (2)
H17B	0.4306	0.0653	0.1877	0.101*	0.54 (2)
H17C	0.4262	−0.1063	0.1344	0.101*	0.54 (2)
C2B	0.3583 (6)	0.041 (3)	0.1485 (9)	0.029 (4)*	0.46 (2)
H2B	0.3679	0.1637	0.1437	0.034*	0.46 (2)
C17B	0.418 (3)	−0.071 (6)	0.184 (4)	0.046 (11)*	0.46 (2)
H17D	0.4385	−0.0718	0.2485	0.069*	0.46 (2)
H17E	0.4442	−0.0207	0.1630	0.069*	0.46 (2)
H17F	0.4084	−0.1885	0.1622	0.069*	0.46 (2)

### Atomic displacement parameters (Å^2^)

**Table:**

	*U*^11^	*U*^22^	*U*^33^	*U*^12^	*U*^13^	*U*^23^
V1	0.0346 (5)	0.0226 (5)	0.0201 (5)	0.0047 (5)	0.0121 (4)	0.0020 (4)
O1	0.052 (3)	0.026 (2)	0.049 (3)	0.000 (2)	0.024 (2)	0.0016 (19)
N1	0.044 (3)	0.024 (3)	0.034 (3)	0.004 (2)	0.022 (3)	0.002 (2)
O2	0.042 (2)	0.028 (2)	0.023 (2)	0.0073 (18)	0.0157 (19)	0.0015 (17)
C3	0.035 (4)	0.052 (4)	0.029 (3)	0.014 (3)	0.008 (3)	0.009 (3)
N4	0.032 (3)	0.025 (3)	0.023 (2)	0.007 (2)	0.013 (2)	0.010 (2)
C5	0.045 (3)	0.022 (3)	0.025 (3)	−0.003 (3)	0.017 (3)	0.003 (3)
C6	0.043 (3)	0.021 (3)	0.032 (3)	−0.001 (3)	0.022 (3)	0.002 (3)
C7	0.053 (4)	0.026 (3)	0.036 (3)	−0.001 (3)	0.027 (3)	0.001 (3)
C8	0.054 (4)	0.040 (4)	0.042 (4)	−0.008 (3)	0.033 (3)	−0.001 (3)
C9	0.039 (4)	0.032 (3)	0.047 (4)	−0.008 (3)	0.023 (3)	−0.008 (3)
C10	0.037 (4)	0.043 (4)	0.069 (5)	−0.003 (3)	0.030 (4)	−0.001 (4)
C11	0.028 (4)	0.052 (5)	0.074 (6)	0.001 (3)	0.012 (4)	−0.006 (4)
C12	0.042 (4)	0.047 (5)	0.063 (5)	0.008 (4)	0.012 (4)	0.014 (4)
C13	0.041 (4)	0.034 (3)	0.041 (4)	0.001 (3)	0.012 (3)	0.004 (3)
C14	0.035 (3)	0.025 (3)	0.035 (3)	−0.005 (3)	0.017 (3)	−0.005 (3)
C15	0.033 (3)	0.019 (3)	0.026 (3)	−0.003 (2)	0.013 (3)	−0.003 (2)
O16	0.036 (2)	0.028 (2)	0.025 (2)	0.0029 (18)	0.0170 (18)	0.0041 (17)
C18	0.052 (4)	0.048 (4)	0.024 (3)	0.006 (3)	0.017 (3)	0.000 (3)
C2A	0.036 (7)	0.042 (10)	0.040 (7)	0.016 (6)	0.011 (6)	0.011 (6)
C17A	0.041 (13)	0.11 (3)	0.051 (13)	0.040 (15)	0.020 (9)	0.037 (15)

### Geometric parameters (Å, °)

**Table:**

V1—O1	1.612 (4)	C9—C14	1.413 (8)
V1—O2^i^	1.658 (4)	C9—C10	1.425 (9)
V1—O16	1.915 (4)	C10—C11	1.358 (11)
V1—N1	2.127 (5)	C10—H10A	0.9300
V1—N4	2.183 (4)	C11—C12	1.382 (10)
V1—O2	2.404 (4)	C11—H11A	0.9300
N1—C2B	1.466 (14)	C12—C13	1.383 (9)
N1—C2A	1.498 (13)	C12—H12A	0.9300
N1—H1A	0.9000	C13—C14	1.401 (9)
N1—H1B	0.9000	C13—H13A	0.9300
O2—V1^i^	1.658 (4)	C14—C15	1.446 (8)
C3—C2B	1.456 (14)	C15—O16	1.320 (6)
C3—N4	1.460 (7)	C18—H18A	0.9600
C3—C2A	1.502 (14)	C18—H18B	0.9600
C3—H3A	0.9700	C18—H18C	0.9600
C3—H3B	0.9700	C2A—C17A	1.52 (6)
N4—C5	1.282 (7)	C2A—H2A	0.9800
C5—C6	1.479 (8)	C17A—H17A	0.9600
C5—C18	1.499 (8)	C17A—H17B	0.9600
C6—C15	1.395 (8)	C17A—H17C	0.9600
C6—C7	1.424 (8)	C2B—C17B	1.59 (6)
C7—C8	1.360 (9)	C2B—H2B	0.9800
C7—H7A	0.9300	C17B—H17D	0.9600
C8—C9	1.413 (9)	C17B—H17E	0.9600
C8—H8A	0.9300	C17B—H17F	0.9600
			
O1—V1—O2^i^	107.6 (2)	C9—C8—H8A	119.6
O1—V1—O16	101.6 (2)	C14—C9—C8	119.4 (6)
O2^i^—V1—O16	100.23 (18)	C14—C9—C10	117.9 (6)
O1—V1—N1	95.9 (2)	C8—C9—C10	122.7 (6)
O2^i^—V1—N1	92.07 (19)	C11—C10—C9	121.1 (7)
O16—V1—N1	154.36 (19)	C11—C10—H10A	119.5
O1—V1—N4	97.7 (2)	C9—C10—H10A	119.5
O2^i^—V1—N4	153.32 (19)	C10—C11—C12	121.2 (7)
O16—V1—N4	82.56 (17)	C10—C11—H11A	119.4
N1—V1—N4	76.61 (18)	C12—C11—H11A	119.4
O1—V1—O2	172.50 (19)	C11—C12—C13	119.3 (7)
O2^i^—V1—O2	77.09 (18)	C11—C12—H12A	120.4
O16—V1—O2	82.98 (15)	C13—C12—H12A	120.4
N1—V1—O2	77.93 (17)	C12—C13—C14	121.5 (7)
N4—V1—O2	76.95 (15)	C12—C13—H13A	119.3
C2B—N1—V1	111.9 (6)	C14—C13—H13A	119.3
C2A—N1—V1	116.4 (5)	C13—C14—C9	119.0 (6)
C2B—N1—H1A	135.2	C13—C14—C15	121.5 (6)
C2A—N1—H1A	108.2	C9—C14—C15	119.5 (5)
V1—N1—H1A	108.2	O16—C15—C6	123.2 (5)
C2B—N1—H1B	78.5	O16—C15—C14	117.4 (5)
C2A—N1—H1B	108.2	C6—C15—C14	119.2 (5)
V1—N1—H1B	108.2	C15—O16—V1	124.1 (3)
H1A—N1—H1B	107.3	C5—C18—H18A	109.5
V1^i^—O2—V1	102.91 (18)	C5—C18—H18B	109.5
C2B—C3—N4	112.9 (7)	H18A—C18—H18B	109.5
N4—C3—C2A	110.7 (6)	C5—C18—H18C	109.5
C2B—C3—H3A	91.7	H18A—C18—H18C	109.5
N4—C3—H3A	109.0	H18B—C18—H18C	109.5
C2A—C3—H3A	122.4	N1—C2A—C3	108.6 (9)
C2B—C3—H3B	124.2	N1—C2A—C17A	111 (2)
N4—C3—H3B	109.1	C3—C2A—C17A	113 (2)
C2A—C3—H3B	96.8	N1—C2A—H2A	108.0
H3A—C3—H3B	107.7	C3—C2A—H2A	108.0
C5—N4—C3	119.9 (5)	C17A—C2A—H2A	108.0
C5—N4—V1	125.8 (4)	C3—C2B—N1	113.0 (10)
C3—N4—V1	114.3 (3)	C3—C2B—C17B	110 (3)
N4—C5—C6	120.8 (5)	N1—C2B—C17B	111 (2)
N4—C5—C18	123.2 (5)	C3—C2B—H2B	106.6
C6—C5—C18	116.0 (5)	N1—C2B—H2B	106.9
C15—C6—C7	119.5 (5)	C17B—C2B—H2B	108.7
C15—C6—C5	121.0 (5)	C2B—C17B—H17D	109.5
C7—C6—C5	119.6 (5)	C2B—C17B—H17E	109.5
C8—C7—C6	121.3 (6)	H17D—C17B—H17E	109.5
C8—C7—H7A	119.3	C2B—C17B—H17F	109.5
C6—C7—H7A	119.3	H17D—C17B—H17F	109.5
C7—C8—C9	120.8 (6)	H17E—C17B—H17F	109.5
C7—C8—H8A	119.6		
			
O1—V1—N1—C2B	−123.4 (9)	C8—C9—C10—C11	179.6 (7)
O2^i^—V1—N1—C2B	128.7 (9)	C9—C10—C11—C12	0.4 (11)
O16—V1—N1—C2B	9.6 (10)	C10—C11—C12—C13	1.3 (12)
N4—V1—N1—C2B	−26.9 (9)	C11—C12—C13—C14	−1.8 (11)
O2—V1—N1—C2B	52.3 (9)	C12—C13—C14—C9	0.6 (10)
O1—V1—N1—C2A	−86.0 (9)	C12—C13—C14—C15	179.0 (6)
O2^i^—V1—N1—C2A	166.1 (9)	C8—C9—C14—C13	180.0 (6)
O16—V1—N1—C2A	47.0 (10)	C10—C9—C14—C13	1.1 (9)
N4—V1—N1—C2A	10.5 (9)	C8—C9—C14—C15	1.5 (9)
O2—V1—N1—C2A	89.8 (9)	C10—C9—C14—C15	−177.4 (6)
O2^i^—V1—O2—V1^i^	0.0	C7—C6—C15—O16	−178.0 (5)
O16—V1—O2—V1^i^	−102.2 (2)	C5—C6—C15—O16	3.1 (8)
N1—V1—O2—V1^i^	95.0 (2)	C7—C6—C15—C14	5.8 (8)
N4—V1—O2—V1^i^	173.8 (2)	C5—C6—C15—C14	−173.2 (5)
C2B—C3—N4—C5	−173.8 (10)	C13—C14—C15—O16	−0.3 (8)
C2A—C3—N4—C5	149.1 (9)	C9—C14—C15—O16	178.1 (5)
C2B—C3—N4—V1	3.9 (10)	C13—C14—C15—C6	176.2 (5)
C2A—C3—N4—V1	−33.1 (9)	C9—C14—C15—C6	−5.4 (8)
O1—V1—N4—C5	−75.5 (5)	C6—C15—O16—V1	45.4 (7)
O2^i^—V1—N4—C5	123.3 (5)	C14—C15—O16—V1	−138.3 (4)
O16—V1—N4—C5	25.3 (5)	O1—V1—O16—C15	47.0 (4)
N1—V1—N4—C5	−169.7 (5)	O2^i^—V1—O16—C15	157.5 (4)
O2—V1—N4—C5	109.8 (5)	N1—V1—O16—C15	−85.1 (6)
O1—V1—N4—C3	106.9 (4)	N4—V1—O16—C15	−49.4 (4)
O2^i^—V1—N4—C3	−54.3 (6)	O2—V1—O16—C15	−127.0 (4)
O16—V1—N4—C3	−152.3 (4)	C2B—N1—C2A—C3	59.9 (13)
N1—V1—N4—C3	12.7 (4)	V1—N1—C2A—C3	−30.7 (14)
O2—V1—N4—C3	−67.8 (4)	C2B—N1—C2A—C17A	−65 (2)
C3—N4—C5—C6	−177.8 (5)	V1—N1—C2A—C17A	−155 (2)
V1—N4—C5—C6	4.7 (8)	C2B—C3—C2A—N1	−60.6 (12)
C3—N4—C5—C18	4.2 (9)	N4—C3—C2A—N1	40.1 (13)
V1—N4—C5—C18	−173.3 (4)	C2B—C3—C2A—C17A	63 (2)
N4—C5—C6—C15	−26.7 (8)	N4—C3—C2A—C17A	164 (2)
C18—C5—C6—C15	151.4 (6)	N4—C3—C2B—N1	−27.1 (16)
N4—C5—C6—C7	154.3 (6)	C2A—C3—C2B—N1	66.4 (14)
C18—C5—C6—C7	−27.6 (8)	N4—C3—C2B—C17B	−152.1 (19)
C15—C6—C7—C8	−2.4 (9)	C2A—C3—C2B—C17B	−59 (2)
C5—C6—C7—C8	176.6 (6)	C2A—N1—C2B—C3	−66.9 (14)
C6—C7—C8—C9	−1.6 (10)	V1—N1—C2B—C3	38.2 (15)
C7—C8—C9—C14	2.0 (10)	C2A—N1—C2B—C17B	58 (3)
C7—C8—C9—C10	−179.2 (6)	V1—N1—C2B—C17B	163 (3)
C14—C9—C10—C11	−1.6 (10)		

Symmetry codes: (i) −*x*+1/2, −*y*+1/2, −*z*.

### Hydrogen-bond geometry (Å, °)

**Table:**

*D*—H···*A*	*D*—H	H···*A*	*D*···*A*	*D*—H···*A*
N1—H1A···O1^ii^	0.90	2.19	3.011 (6)	151
C7—H7A···O2^iii^	0.93	2.41	3.335 (7)	173
C18—H18B···O16^iii^	0.96	2.56	3.482 (8)	161
C3—H3B···Cg1^iii^	0.97	2.95	3.874 (7)	159

Symmetry codes:  (ii) −*x*+1/2, −*y*−1/2, −*z*; (iii) −*x*+1/2, *y*−1/2, −*z*+1/2.

### Table 2 π–π interactions (Å,°).

**Table:**

CgI	CgJ	Cg···Cg	Dihedral angle	Interplanar distance	Offset
Cg2	Cg2^iii^	3.518 (4)	0.0	3.365 (4)	1.025 (4)

Symmetry code: (iii) -x, -y, -z. Notes: Cg2 represents the centre of gravity of the ring C14-C19. Cg···Cg is the distance between ring centroids. The dihedral angle is that between the planes of the rings CgI and CgJ. The interplanar distance is the perpendicular distance of CgI from ring J. The offset is the offset distance of ring I from ring J.
